# Contribution of Developmental Psychology to the Study of Social Interactions: Some Factors in Play, Joint Attention and Joint Action and Implications for Robotics

**DOI:** 10.3389/fpsyg.2018.01992

**Published:** 2018-10-19

**Authors:** Hélène Cochet, Michèle Guidetti

**Affiliations:** CLLE, Université de Toulouse, CNRS, UT2J, Toulouse, France

**Keywords:** human–robot interaction, human development, joint attention, joint action, coordination, complexity, gestures

## Abstract

Children exchange information through multiple modalities, including verbal communication, gestures and social gaze and they gradually learn to plan their behavior and coordinate successfully with their partners. The development of joint attention and joint action, especially in the context of social play, provides rich opportunities for describing the characteristics of interactions that can lead to shared outcomes. In the present work, we argue that human–robot interactions (HRI) can benefit from these developmental studies, through influencing the human’s perception and interpretation of the robot’s behavior. We thus endeavor to describe some components that could be implemented in the robot to strengthen the feeling of dealing with a social agent, and therefore improve the success of collaborative tasks. Focusing in particular on motor precision, coordination, and anticipatory planning, we discuss the question of complexity in HRI. In the context of joint activities, we highlight the necessity of (1) considering multiple speech acts involving multimodal communication (both verbal and non-verbal signals), and (2) analyzing separately the forms and functions of communication. Finally, we examine some challenges related to robot competencies, such as the issue of language and symbol grounding, which might be tackled by bringing together expertise of researchers in developmental psychology and robotics.

## Introduction

Developmental psychologists aim at describing and explaining changes across the life span in a wide range of areas such as social, emotional, and cognitive abilities. Focusing on childhood is a way of grasping numerous changes, especially in terms protect of communication: infants gradually learn to identify the common ground they have with others and engage in social interactions. The development of such abilities relies on the personal experiences shared between partners in specific contexts (Liebal et al., 2013), among which social play may offer particularly rich opportunities for children to acquire joint action and joint attention skills. Studying the different forms and functions of communication in this context paves the way for identifying the necessary ingredients for effective joint activities and therefore better understanding the architecture of human–social interactions. Even though the concept of *effectiveness* may cover different theoretical frameworks, the latter objectives have several applications, for example in supporting children with atypical development, especially when they have difficulty communicating both verbally and non-verbally (e.g., children with autism spectrum disorders, ASD), but also in the field of artificial intelligence. The role of robots in society raises indeed a lot of debates and challenges, as they share more and more space and tasks with humans, for instance in service robotics to assist elderly people. The robots’ ability to initiate and respond to social interactions is one of the key factors that will shape their integration in our everyday life in the future. Researchers in social robotics have been working on the question of joint action for over two decades now, sometimes in collaboration with developmental psychologists (e.g., Scassellati, 2000), in order to improve robots’ motor and communicative skills. Developmental models of human communicative behavior can indeed help define the components to implement in human–robot interactions (HRI), so as to build rich and natural joint activities (Breazeal et al., 2004; Lemaignan et al., 2017).

The objective of this paper is twofold. First, we intend to present the point of view and some research perspectives of developmental psychologists on joint attention and joint action, in particular in the context of social play. To this end, we will also define, starting from studies on non-human primates, what can be regarded as complex (or rich) and natural (or effective) interactions in both human communication and HRI. Second, we aim to show the extent to which the above-mentioned issues may be of interest to roboticists, in helping conceptualize and implement some variables associated with joint attention and joint action in the context of HRI. Collaborative tasks involving robot and human partners, regarded as tantamount to children’s social play, will thus be considered through the prism of pragmatic communication, allowing researchers to dissociate the forms and the functions of communication.

## How Does Communication Develop in the Context of Social Play?

The definitions of play include a wide range of activities, which makes it difficult to determine where play begins and where it ends, even though it is traditionally associated with positive affective valence (Garvey, 1990). Play, which occurs in several animal species (most notably in mammals), has been argued to allow “practice of real-world skills in a relatively safe environment” (Byrne, 2015). We will focus here on social play in human children, which may also enable them, as highlighted by Bruner (1973), to “learn by doing” as they interact with one or several partners. At the individual level, children can indeed explore and enhance specific skills like motor control and creativity, while developing for example cooperation abilities at the social level. The concepts of artifact-mediated and object-oriented action, originally formulated by Vygotsky (1999), are particularly relevant to describe these situations: the relationship between the child and the surrounding objects is indeed mediated by cultural means, tools, and signs. Studying the development of play can therefore reveal how children come to represent and think about their environment.

Social attention is a crucial capacity for the emergence of these play situations, allowing children to focus on some of the other’s characteristics such as the facial expressions, gaze direction, gestures, and vocalizations. When the direction of another’s attention has been identified (for example through gaze following or point following), we can shift our own attention to focus at the same time on the same external object or event as our partner. This process of *joint attention* is usually inferred from behavioral cues, including mainly gaze alternation between one’s partner and a specific referent (Bourjade, 2017). Joint attention seems therefore necessary for individuals to perform joint action, i.e., to coordinate their actions in space and time to produce a joint outcome, whether it involves here symbolic play (with or without objects), construction toys, board games or any other forms of play.

Joint attention and joint action begin to appear at the end of the first year in human development (Carpenter et al., 1998), gradually allowing children to integrate the notion of common ground and engage in social interactions. The development of gaze understanding, which has been widely studied, plays a key role in this regard. It was for example shown in a study using habituation-of-looking-time procedure that infants start to understand ecologically valid instances of social gaze between two adults interacting, and to have expectations concerning gaze target at 10 months of age (Beier and Spelke, 2012). Besides, responsive joint attention skills (e.g., gaze following and point following) have been reported to emerge before initiative joint attention skills, from 8 months of age (Corkum and Moore, 1998; Beuker et al., 2013).

However, depending on the authors, the definitions of these social-cognitive skills can be more or less demanding, the main difference lying in whether or not individuals have mutual understanding of their shared focus of attention. The ability to “know together” that we are attending to the same thing as our partner has sometimes been referred to as *shared attention* (Emery, 2000; Shteynberg, 2015), which would develop in parallel with *shared intentionality* (Tomasello and Carpenter, 2007). The latter involves the motivation to share goals and intentions with the other, as well as forms of cognitive representation for doing so. This ability has been argued to constitute a hallmark of the human species (Tomasello et al., 2005), even though it is particularly difficult to assess when verbal language is not available as a clue to these representations (in pre-linguistic children or non-human primates). Similarly, joint action may rely solely on the learning of the cues that appear significant (e.g., gestures and eye contact) to coordinate actions in space and time with a partner, or it may also involve, in a more demanding perspective, the common and explicit knowledge of the objectives of the activity and of the way to achieve them (Tomasello and Carpenter, 2007).

Joint attention and joint action, whether they are accompanied or not with shared and explicit intentions, thus allow children to participate with others in collaborative activities in which each partner benefits from the joint outcome and/or from the interaction in itself. In a series of experiments, the ability to coordinate with a partner in social games was shown to significantly improve between 18 and 24 months of age, whether the games involved complementary or similar roles (Warneken et al., 2006). In the first game of this study, one person had to send a wooden block down one of a tube mounted on a box on a 20 degrees incline, while the other person had to catch it at the other end with a tin can that made a rattling sound. Two tubes were mounted in parallel so that individuals could perform in turn the different roles. In the second game, two persons had to make a wooden block jump on a small trampoline (67 cm diameter ring covered with cloth) by holding the rim on opposite sides. The trampoline collapsed when being held on only one side. Children successfully participated in both games, although the 24 month-olds were more proficient than the 18 month-olds, and they all produced at least one communicative attempt to reengage the adult partner when the latter stopped participating in the activity. Children for example pointed at the object, and/or vocalized while looking at the adult, which was regarded as evidence for a uniquely human form of cooperation, involving shared intentionality (Warneken et al., 2006). A less “mentalistic” interpretation could be proposed (D’Entremont and Seamans, 2007), but these results nevertheless highlight children’s motivation for reinstating joint action toward a shared goal. The development of this capacity has received much attention from researchers, as the initiation of joint attention appears to be strongly related to language comprehension and production in the second and third year of life (Colonnesi et al., 2010; Cochet and Byrne, 2016), as well as to theory of mind ability (e.g., Charman et al., 2000; Milward et al., 2017) in both typical and atypical development (e.g., Adamson et al., 2017).

In addition, the observation of children’s behavior during collaborative activities may lead to a thorough description of multimodal communication (e.g., gaze, facial expressions, gestures, and verbal language) and of the way its components become coordinated. For example, the production of gestures gradually coordinates with gaze in the course of development. Children start to produce pointing gestures to orient the attention of another person around 12 months of age; an object, a person or an event can become the shared focus of attention but then children do not usually look at their partner while they point (Franco and Butterworth, 1996). A couple of months later, they are able to alternate their gaze between their partner and the object of interest, which represents a key feature of intentional triadic interactions (Cochet and Vauclair, 2010). At 16 months of age, gaze toward the adult can precede the production of pointing (Franco and Butterworth, 1996), suggesting that children may thus take into account the partner’s attentional state before initiating communication (Lamaury et al., 2017).

Children also gradually learn to take account of their partner’s facial expressions to infer their emotional state and adjust their response accordingly. Infants are sensitive to the characteristics of faces from very early on; newborns look for example significantly longer at happy expressions than at fearful ones, demonstrating some discrimination skills (Farroni et al., 2007). The still-face paradigm, initially designed by Tronick et al. (1978) also suggests that infants have expectations about interactional reciprocity from a few months of age, partly relying on emotional expression. This sensitivity manifests itself in specific behavioral and physiological responses (e.g., reduced positive affect and gazing at the parent, increased negative affect, rise in facial skin temperature) when the mother puts on a neutral and unresponsive face, after a period of spontaneous play with his/her infant (Aureli et al., 2015). The ability to recognize and identify facial expressions of basic emotions further develops in preschool children, before they can understand a few months later the external causes of emotions and then, around 5 years of age, the role of other’s desires or beliefs in emotional expression (Pons et al., 2004).

During play interactions, being attentive to the other’s facial expressions allows each partner to consider the emotional nature of the signals (e.g., joy, surprise, and frustration) and to possibly modify his/her own behavior to change or maintain this emotional state. The development of facial expression perception thus plays a key role in the emergence of joint actions, in coordination with other communicative modalities. Facial expressions are indeed usually synchronized with vocalizations and/or gestures, and this from infancy.

The vocal and the gestural modalities also become more and more coordinated as children grow older, which represents a key feature of human communication as we use gestures as we speak throughout our life. Communicative gestures are first complemented by vocalizations, whose prosodic patterns may already code for semantic and pragmatic functions (Leroy et al., 2009). In the second year of life, children then produce their first gesture-word combinations, which have an important role in the transition to the two-word stage (e.g., Butcher and Goldin-Meadow, 2000). Pointing and conventional gestures (e.g., waving goodbye, gestural agreement, and refusal: Guidetti, 2002, 2005) remain in the child repertory after the two-word stage, but other forms of gestural-vocal coordination are observed from 3 years of age with the emergence of co-speech gestures. Although we are usually not aware of producing or perceiving them, co-speech gestures can lend rhythm, emphasize speech and sometimes serve deictic or iconic functions. The deictic presentation of pointing gesture can for example be combined with *vocal pointing*, performed through syntactic or prosodic means (Lœvenbruck et al., 2008). Such coordination between the vocal and gestural modalities is omnipresent in adults and play a crucial role in face-to-face communication for both speaker and listener (e.g., McNeill, 2000; Kendon, 2004).

Moreover, the characteristics of gaze, gestures, and vocalizations and their coordination may vary according to the communicative function of the signal. A gesture can indeed serve different purposes, starting with the traditional distinction between imperative and declarative functions (Bates et al., 1975). Imperative gestures are used to request a specific object or action from a partner whereas declarative gestures are used to share interest with the other about some referent or provide him/her with information that might be useful. Imperative and declarative pointing, which both represent powerful means of establishing joint attention, have been extensively studied and compared: hand shape and body posture were shown to differ according to the communicative function of the pointing gesture (Cochet et al., 2014), as well as the frequency of gaze alternation between the partner and the referent and the frequency of vocalizations (Cochet and Vauclair, 2010). These comparisons (see section “Pragmatics in HRI: Which Ingredients Are Necessary for Effective Interactions?” for more detailed results) thus highlight the strong relationship between the form of the gestures (in the broad sense, i.e., including visual and vocal behavior in addition to movement kinematics and hand shapes) and pragmatic features in children, even semantic ones in adults (Cochet and Vauclair, 2014).

To sum up, when two children are playing together or when a child is playing with an adult, they do so in the framework of joint action; they attend to a common situation and use multimodal communication to initiate, maintain, or respond to the interaction. These three different roles in the interaction can be assessed with the Early Social Communication Scales, in particular with the French version (Guidetti and Tourrette, 2017). In an evaluation situation, giving the child the opportunity to initiate the interaction is particularly crucial in atypical development, for example in children with ASD. The initiation of shared attention is a key ability in this context as it allows joint action coordination (Vesper et al., 2016) and has also significant consequences on the development of cognitive and emotional processes (Shteynberg, 2015). Whether this coordination relies on the representation and the understanding of the other’s intentions or only on behavioral cues is a challenging question, as we do not have any direct access to the other’s subjectivity. In the field of HRI, an objective that appears sufficiently ambitious for now, or at least the one we chose to focus on in the present review, is to design robots able to identify the observable changes in the human’s behavior, in order to make the right inferences and thus the appropriate decisions in the interaction. This appears as an essential condition for a successful exchange between a robot and a human, which can depend on the joint outcome (has the common goal been reached?), but also on the way the interaction has been perceived by each individual, for example in terms of coordination between gaze and gesture and fluidity of movement (Hough and Schlangen, 2016). The richness of communication here lies indeed in the ability of each partner to integrate multiple communicative cues in a way that what will seem *natural* to the humans, i.e., that will be close to peer interaction in everyday life.

This appears as a complex ability and probably the most challenging one to replicate in HRI. In pursuit of this objective, we now need to further describe the concept of *appropriateness* and propose a frame to determine the relative importance and the relative complexity of the different behaviors observed during joint activities such as social play.

## To What Extent Can Interactions Be Characterized as *Complex*?

Smith (2015) has argued that “development, like evolution and culture, is a process that creates complexity by accumulating change.” This perspective applies to the development of social interactions, from the emergence of joint attention to coordinated and multimodal communication that enable joint action. Several attempts have been made in developmental robotics to explore the cognitive, social, and motivational dynamics of human interactions (Oudeyer, 2017); algorithmic and robotic models can then be used to study the developmental processes involved for instance in imitation (Demiris and Meltzoff, 2008) or language (Cangelosi et al., 2010). In this context, roboticists aim at designing systems allowing for self-organized and “progressive increase in the complexity” of the robot’s behavior (Oudeyer et al., 2007).

To benefit further from their exchanges, developmentalists and roboticists may therefore need to frame the study of HRI by disambiguating the concept of complexity. Because “complicated systems will be best understood at the lowest possible level” (Smith, 2015), we aim to differentiate different levels of complexity depending on the nature of the elements to take into account for decision making. This analysis will allow us to go forward in the study of joint attention and joint action and define what is implied by the qualifying terms “complex” (or rich) and “appropriate” (or effective) when referring to interactions.

To this end, we used a categorization recently proposed in research on animal behavior, including human and non-human primates, to define the concept of complexity (Cochet and Byrne, 2015). Three dimensions have been described: motor precision, coordination, and anticipatory planning, which can relate to both individual and social activities. The authors argue that “the complexity of a given mechanism/behavior can be assessed by distinguishing which of these three dimensions are involved and to what degree,” which may “clarify our understanding of animal behavior and cognition.” Such analysis applied to joint attention and joint action, although there may be other ways of untangling the question of complexity, may here allow researchers to dissect the different factors involved in social interactions for each dimension, and thus help them assess the “manipulability” of these factors in HRI.

In order to make appropriate decisions in a collaborative task, i.e., decisions leading to the desired joint outcome and/or decisions that approach the characteristics of human interactions, the robot first needs to recognize specific patterns in his/her partners’ behavior, without asking for agreement or information for all actions. The robot can for example rely on gaze direction, manual movements or body posture to identify the human’s attentional and intentional states and thus define the most useful role it can play in the interaction. By way of illustration, if a human and a robot share the common goal of building a pile with four cubes in a definite order and putting a triangle at the top, each of them can perform different actions: they can grasp an object (a cube or a triangle) on the table, grasp an object on the pile, give an object to the partner, support the pile while the partner places a cube on it, etc. Other actions can emerge, for example if the pile collapses or if one agent does not pile the cubes in the correct order (Clodic et al., 2014). Individuals can then blame each other, or give each other some instructions. In addition to the perception of its own environment, the robot thus has to observe the activity of the human and take his/her perspective (e.g., to determine whether an object is reachable for the other).

Motor precision is therefore necessary in this context to obtain flexible and human-aware shared plan execution (Devin and Alami, 2016), as it enables a selective shift of attention toward aspects of the environment that will become shared knowledge, which has also been described as the *accuracy* of shared attention states (Shteynberg, 2015). First, the emergence of joint attention requires to properly use gaze and/or pointing gesture to localize the object or event referred to. Verbal cues also demand particularly fine motor skills through speech articulators. Second, joint action necessitates some motor control to reach the expected outcome, hence the importance of evaluating beforehand human motor skills, especially during development, as well as the technical capabilities of the robot. Following on from the previous example, children’s grasping skills in relation to the size of the cubes as well as the characteristics of robotic gripper to handle objects have to be finely described.

Moreover, recent experimental findings have shown that the execution of object-oriented actions is influenced by the social context such as the relative position of another person and the degree of familiarity with this person (Gianelli et al., 2013). Individuals perform for example more fluent reach-to-grasp movements, with lower acceleration peaks and longer reaction time when a partner is located close enough to be able to intervene on the same object than when he/she is farther away (Quesque et al., 2013). In addition, there is a significant relationship between the kinematic features of the actions and the actor’s explicit social intention: movements have longer durations, higher elevations and longer reaction times when individuals place an object on a table for another person than when they place the object for a later personal use (Quesque and Coello, 2015). These variations, although they do not seem to be intentionally produced, have been suggested to facilitate the partner’s detection of planned actions, thus enhancing potential interactions. These kinematic effects were indeed shown to influence the subsequent motor productions of an observer (Quesque et al., 2015). The motor characteristics of actions performed in a social context may therefore prime the perceiver to prepare and anticipate appropriate motor responses in the interaction.

The second dimension that can allow us to understand the complexity of joint activities pertains to the coordination between several communicative modalities and between interacting individuals. Whether joint action involves complementary or similar roles, it can be performed through several coordination processes, which can determine the *efficiency* of shared attention states (Shteynberg, 2015). Efficiency requires here a representational shift from the first-person singular to the first-person plural, as the partners attend to the same referent at the same time. The ability to monitor each other’s attention and action, using behavioral cues such as gaze direction, facial expressions, gestures, and speech is essential for successful coordination. The intentional production of communicative signals, representing hints for one’s partner, is also an efficient way of achieving joint outcomes.

Coordination is therefore necessary first at the individual level, so that the different communicative modalities such as gestures and gaze synchronize or follow one another in a natural order, i.e., acceptable with regard to human interaction patterns (see above). Each agent can then make decisions based on these signals, moderate their behavior accordingly and thus coordinate at the social level to reach a common objective. The ability to adjust one’s behavior to others’ actions during collaborative activities (including play) has been argued to “reach a higher degree of complexity when intentional and referential signals are directly addressed to specific individuals” (Cochet and Byrne, 2015). In order to build the pile of cubes, interacting partners can then for example point toward a specific cube or ask the other to wait before placing another cube.

In those cases, coordination processes can be enhanced by predicting the effects of each other’s actions on joint outcomes and by distributing tasks effectively (Vesper et al., 2016). This ability involves the third dimension characterizing the question of complexity, namely the dimension of anticipatory planning (Cochet and Byrne, 2015). It requires to go beyond the immediate perception of the environment and represent the relationship between a sequence of actions and a precise goal. At the individual level, planning ability implies to mentally review an action sequence in anticipation of a future need (e.g., selecting a specific cube in a first room in order to build a pile of cubes in another room). At the social level, planning ability allows individuals to predict the other’s behavior and adjust one’s own sequence of actions, leading to a better coordination. Whether the ability to make such inferences necessitates to mentalize about others’ inner states (e.g., beliefs and preferences) is still subject of debate, but again, this question may not be central in the context of joint attention and joint action between a robot and a human.

The above-described categorization can therefore provide a common ground between ethologists, psychologists, and roboticists that may clarify which dimensions need to be considered in an attempt to implement the characteristics of motor precision, coordination and anticipatory planning in human–robot joint activities (see **Table 1** for an overview). The objective is to approach the complexity (or richness) of human interactions and obtain appropriate (or effective) responses from robots with regard to these different dimensions.

**Table 1:** Complexity in HRI: illustration of three dimensions at the individual and social levels (adapted from Cochet and Byrne, 2015).

	Individual	Social
(1) Motor precision	Joint attention: ability to properly use gaze or pointing to identify the object or event referred to. Joint action: human motor skills/technical capabilities of the robot to reach the expected outcome	Influence of the social context (e.g., relative position of the individuals, intention of the actor: moving an object for oneself or for another person) on the kinematics features of the actions performed
(2) Coordination	Coordination (including synchronization) between different modalities of one’s communicative signal (gaze, gesture, vocalizations, etc.)	Ability to take into account the multimodal behavioral cues produced by a partner to adjust one’s own behavior
(3) Anticipatory planning	Representation of a sequence of actions to anticipate a personal future need	Ability to predict the effects of the other’s actions on joint outcomes to plan one’s own behavior


## Pragmatics in HRI: Which Ingredients Are Necessary for Effective Interactions?

The increasing complexity of communicative abilities (complexity that involves the three above-mentioned dimensions) in the course of human development leads to a rich potential of interactions. Children actively go through different stages allowing them to engage successfully in joint activities, i.e., to operate within their physical environment, coordinate with other people, plan their own behavior and anticipate their partners’. Intending to model, at least partially, human developmental pathway seems a fruitful way of designing robots that can effectively initiate and respond to communicative situations. Such enterprise, although still recent, has given rise to a substantial amount of literature in robotics, especially from the 2000s, covering several sub-fields such as for example developmental and epigenetic robotics, cognitive systems and social robotics. Several journals, including both HRI experimental studies and computational modeling, focus entirely on these questions (e.g., *IEEE Transactions on Cognitive and Developmental Systems,* *Journal of Human-Robot Interaction, Journal of Social Robotics*), and numerous conferences also take place every year, whose proceedings are usually available online^1^.

The data from developmental psychology described in the first section, coupled with the framework proposed in the second section to help researchers define complex and effective HRI, may contribute to this growing body of work. To this effect, it seems necessary (1) to consider the multimodality of interactions and (2) to adopt a pragmatic perspective to be based upon an accurate representation of human communicative behaviors. Indeed, children learn to communicate through joint activities with adults who combine various forms of expressions, serving various functions. In the course of development, children gradually integrate the dissociation between the form and the function of language – they become more and more flexible in understanding that a single form can serve different functions and reciprocally, that a single function can be expressed through several forms. Language is here regarded as more than a medium to convey an information, in agreement with a proposition that was developed in the speech act theory (Austin, 1962; Searle and Vanderveken, 1985). Language would be way of acting on the environment, of “doing things with words,” independently of its structural properties. Initially aiming at describing the relationships between the forms and functions of linguistic utterances, this theory defines several speech acts, depending on whether one intends to assert, comment, warn, request, deplore, etc. This theory has later been adapted to non-verbal behavior (e.g., McNeill, 1998; Guidetti, 2002). The form still refers to the message structure, but applies to the whole body, including the posture, the structure of communicative gestures (kinematic features and hand shape), gaze and facial expressions. These non-verbal signals can be used in complementarity with speech or be used alone for example in the case of conventional gestures (see Guidetti, 2002). The function refers to the illocutionary force of the speech act (what one achieves by speaking), in other words here to the effect of these communicative acts in a specific context, thus giving some insight into the signaller’s intention. Gestures, and especially the conventional gestures produced by children during the prelinguistic period, are thus regarded as genuine communicative acts, with a propositional content that can equal the one expressed by words. For instance, agreeing and refusing can be expressed gesturally by nodding or shaking one’s head. The separate analysis of the forms and functions of communication, as well as the description of the different modalities involved during interactions, therefore provide a key framework to help define what capacities the robot should be equipped with to ensure efficient collaboration with humans.

In this perspective, Mavridis (2015) has proposed a list of “ten desiderata that human–robot systems should fulfill” to maximize communication effectiveness. One of the guiding lines relates to the importance of considering multiple speech acts, for both verbal and non-verbal communication, and not restrict the robot competencies to “motor command requests.” In the same way as imperative gestures (see section “How Does Communication Develop in the Context of Social Play?”) are generally understood and produced later than declarative gestures in human development (Camaioni et al., 2004), robotic systems initially aimed to assign the robot a servant role, with the human driving the interaction. Devising wider robots’ pragmatic abilities is a first step toward the conception of human–robot shared plans. The robot may for example comment on the pile of cubes as it is being built (see example section “To What Extent Can Interactions Be Characterized as *complex*?”) to support or correct the human’s action, rather than just producing a motor response to the human request. The dimension of social coordination is thus added to that of motor precision (see **Table 1**).

Similarly, flexibility in HRI also requires “mixed initiative dialog” (Mavridis, 2015), so that the robot can both initiate and respond to the interaction. Integrating models based on human adaptation and probabilistic decision processes, Nikolaidis et al. (2017) have indeed shown that the performance of human–robot teams in collaborative tasks is improved when the robot guides the human toward an effective strategy, compared to the common approach of having the robot strictly adapting to the human. The human’s trust in the robot was also facilitated by a greater symmetry in role distribution and adaptation between the robot and the human, which might in turn lead to greater acceptability of HRI.

Designing such “socially intelligent and cooperative robots” (Breazeal et al., 2004) requires specific temporal dynamics of the interaction, which represents a considerable challenge especially at a computational level. These dynamics convey social meanings to such an extent that any delay in the interaction can sometimes question its effectiveness. Researchers here face a dilemma that seem to bring into opposition interaction complexity (which requires to take account of numerous parameters) and interaction timing. The implementation of fast timescales (on the order of 100 ms) is usually considered necessary for robots to integrate (i.e., detect, interpret, and predict) and react to social stimuli in a timely manner through interactions (Durantin et al., 2017). Researchers developing a storytelling robot interacting with children aged 4–5 years have confirmed the importance of temporal features in the pragmatics of interactions. Contingent responses from the robot, in relation to the attentional and social cues signaled by the children, were indeed found to facilitate engagement of the latter (Heath et al., 2017).

The variation in some characteristics of the robot’s behaviors according to the action performed may also illustrate further the question of pragmatics in HRI, moving us one step closer toward human-like interactions. For example, the morphological differences that have been reported in young children between pointing and reaching (Cochet et al., 2014) could be applied to the robot. First, regarding body posture, we might expect robots to lean closer to a given object when they intend to grasp it than when they want to communicate about that object. Second, depending on the robot technical possibilities (e.g., two- or three-finger grippers, biomimetic anthropomorphic hands), differences in the form of manual gestures produced should be observed between imperative and declarative pointing. The former is typically characterized by whole-hand gestures (all the fingers are extended in the direction of the referent), while the latter is mostly associated with index-finger gestures (the index finger is extended toward the referent and the other fingers are curled inside the hand) (Cochet and Vauclair, 2010; Liszkowski and Tomasello, 2011). Hand shape is also influenced by precision constraints: imperative gestures are likely to shift from whole-hand pointing to index-finger pointing when the target is surrounded by distractors (Cochet et al., 2014), which can be the case when the robot has to identify a specific object among several (e.g., the human can ask the robot to give him/her the red cube). Here, the notion of iconicity, which plays a role in both oral and sign languages, may help researchers to precisely analyze the structure of gestures and better understand the interface between gestures and signs (Guidetti and Morgenstern, 2017). The importance of motor precision is here directly related to the dimensions of coordination and anticipatory planning, therefore providing a comprehensive framework to assess the complexity and effectiveness of HRI.

Moreover, the importance of implementing responsive social gaze in robots has previously been highlighted (e.g., Yoshikawa et al., 2006), but this response might also vary depending on the communicative function involved. To mirror child development, gaze alternation between the partner and the referent should indeed be more frequent in declarative situations than in imperative ones (Cochet and Vauclair, 2010). The coordination between gestures and gaze (see also section “How Does Communication Develop in the Context of Social Play?”) is also an important factor, which can help the robot to estimate the state of goals, plans, and actions from human point of view, and allow the human to feel that he/she is involved in fluid interactions with the robot, both facilitating the emergence of joint outcomes. If a robot alternates its gaze between an object and its partner before initiating a pointing gesture, the human may for example interpret this behavior as the robot’s willingness to take into account his/her attentional state before gesturing, thus favoring the exchange of information. Broadly speaking, coordinated gaze behavior could be considered as the most fundamental modality for effective HRI, or at least as a key prerequisite in collaborative tasks.

The consideration of facial expressions may also facilitate turn-taking dynamics and limit miscommunication, by allowing some inferences about the other’s affective state. Integrating the emotional component into HRI gives each partner additional cues to decide what is the most appropriate response in a given situation. The development of methods for facial expression analysis raises several issues though (e.g., Kanade et al., 2000). Even if there have been some attempts to design facial expression mechanism in humanoid robots (e.g., Hashimoto et al., 2006; Gao et al., 2010), most of current robots’ facial features are still far from the extremely rich motor possibilities of the human face. In parallel, the development of real time coding of emotional expressions seems to be an achievable goal (Bartlett et al., 2003), allowing robots to directly perceive some changes in the human facial expressions.

In addition to visual information, the auditory modality can also play a role in influencing robots’ and humans’ decisions and coordination processes. In children at around 2 years of age, vocalizations accompany more frequently declarative gestures than imperative ones (Cochet and Vauclair, 2010). More recently, the prosody of these vocalizations was shown to gradually match the function of pointing during the second year of life (Tiziana et al., 2017), allowing to differentiate imperative from declarative gestures (Grünloh and Liszkowski, 2015). Other features such as the positioning of the object and the attentional state of the partner have also been suggested to influence the rising and falling tones in the vocal productions simultaneous to gestures (Leroy et al., 2009). Prosody can therefore serve pragmatic purposes, and changes in pitch, intensity, or duration of speech or vocalizations can in this regard be considered as a full-fledged component of multimodal communication.

Beyond prosody, language content may be the most effective way for human–robot teams to coordinate. However, the design of robots with language comprehension and production abilities that could lead to fluid conversations with humans raises several issues. Verbal language requires indeed symbolic representations, which need to be connected not only to the robot’s sensory system, but also to “mental models” of the world internalized within its cognitive system. Mavridis (2015) has highlighted here the question of “situated language and symbol grounding.” For example, the relation between the verbal label “cube” uttered by the human and the physical cube that it refers to in front of the robot can be mediated through sensory data, but the use of conventional signs should allow the robots to go beyond the *here-and-now* and extend symbol grounding to abstract entities in addition to objects, people, or events. To implement architecture that can be compared to human interactions, this relation should be bidirectional: the visual perception of a cube should activate the right symbol in the robot’s cognitive system, leading to the production of the word “cube”; reciprocally, a request addressed to the robot to give the human the cube should create a precise representation, allowing the robot to identify the right object.

Moreover, the identification of emotion labels in the verbal modality could also contribute, in addition to the recognition of emotional facial expressions and acoustic properties of speech (see Breazeal, 2004 for a complete review on emotion systems in robots), to a better coordination between each partner of the interaction. The haptic modality, playing an important role in social interactions, is also regarded as a valuable medium for expressing emotion (Yohanan and MacLean, 2012). By developing motion capturing system and tactile sensors, the robot may use its human partner’s positions and such “affective touch” to estimate human intentions (Miyashita et al., 2005). This modality, essential in human development, may be a particularly good candidate to study complexity of HRI, involving simultaneously motor precision, coordination and planning (see section “To What Extent Can Interactions Be Characterized as *complex*?”).

Finally, in addition to the coordination dimension, the verbal dialog between a robot and a human would ideally imply purposeful speech and planning (Mavridis, 2015), in order to avoid fixed mapping between stimuli and responses. Anticipatory planning abilities, as described in Section “To What Extent Can Interactions Be Characterized as *complex*?”, would enable the robot to make the most appropriate or efficient decisions in a given shared activity, in conjunction with its perspective-taking skills and the goal of the activity. If the robot can represent which information are needed by the human to perform a specific action (and therefore identify which information the human misses), it can decide to express a verbal request or comment on the situation, and/or plan a sequence of actions to coordinate with its partner.

This last example raises the question of intrinsic motivation in interactions: why is each partner engaged in this multimodal coordination, and to what extent does it influence the characteristics of the interaction? Studies in developmental robotics have shown that intrinsic motivation systems based on curiosity can directly impact learning skills and lead to autonomous mental development in robots (Oudeyer et al., 2007). Such mechanism is obviously involved in human development and in social play in particular: children discover and create new possibilities by exploring their physical and social environment. Through the development of social referencing, self-consciousness or cooperation, human social interactions may even sometimes constitute a motivated goal *per se* (Tomasello, 2009), which provides some perspectives to shape robots’ intrinsic motivation with a “social reward” function.

We can see here that the relationships between theories in developmental psychology and robotics offer bidirectional benefits. To put it in a nutshell, some models in developmental robotics are based on psychological theories, which are then formalized and implemented in robots, while developmental robotics allows researchers in psychology to go further in the elaboration of their theories through thorough experimentations and hypothesis testing. This applies to a variety of questions addressed in this review, from the conditions that influence learning process during interactions (Boucenna et al., 2014) to the description of stages in language development (Morse and Cangelosi, 2017). Advances in developmental robotics may thus provide previous help in the analysis and implementation of the processes involved in interactions.

## Conclusion and Perspectives

The question at stake in the present work was to improve the effectiveness of human–robot interactions in collaborative tasks, first in terms of joint outcomes – has the task been completed? – but also with regard to the human’s perception and interpretation of the interaction. Is the robot’s behavior appropriate, i.e., acceptable, considering the frame of human communication? We argue here that the observation of the development and the structure of interactions between the child and the adult, especially in the context of social play, can help answer this question. To shape a shared common space between the human and the robot that could reflect the complexity of human interactions, we have also proposed to focus on three dimensions: motor precision, coordination, and anticipatory planning. The specific examples developed in Section “Pragmatics in HRI: Which Ingredients Are Necessary for Effective Interactions?” suggest that the more robots use human-like communicative modalities (e.g., facial expressions, gestures, and language) in respect to these three dimensions, the more they invite interactive behaviors that are natural to people. The interpretation of dealing with a social agent is strengthened, which facilitates in turn the interaction with robots. In this sense, and to paraphrase Cangelosi et al. (2010), the integration of action and language may constitute a roadmap to better frame and assess HRI from a developmental point of view and with a pragmatic perspective.

However, there are still numerous obstacles before achieving the level of details pictured in the present article, involving mainly technological challenges, given the motor and cognitive correlates of the above-mentioned behaviors. To put it bluntly, developmental psychologists cannot expect roboticists to implement in robots all the subtleties of multimodal communication that occur in human children. There may also be some conceptual difficulties as the attempts to approach human realism, aiming at maintaining the human’s trust in the robot, can sometimes be confronted with an uneasy feeling of viewing and/or hearing a robot that looks imperfectly human. This *uncanny valley* effect (Mitchell et al., 2011; Mori, 1970, 2012), which was shown to emerge in middle childhood in relation to developing expectations about humans and machines (Brink et al., 2017), may complicate the design of socially interactive robots, both in terms of appearance and behavior. Empirical evidence for the uncanny valley seems nevertheless inconsistent or restricted to specific conditions (Kätsyri et al., 2015), with the definition of human-likeness mostly involving physical realism.

By contrast, anthropomorphic behavior (see Duffy, 2003), in addition to its facilitating role in the interaction with humans (see above), also results in better and faster learning by the robots. For example, in a task in which they have to learn the meaning of words, the robots’ performances are enhanced when they provide humans with social cues to communicate a learning preference, as these cues influence the tutoring of the human teacher (de Greeff and Belpaeme, 2015). We observe the same phenomena when human children start to learn new concepts: according to Bruner’s constructivist theory, children need scaffolding from adults (or from children who have already acquired the concept) in the form of active support, which may represent at first a reduction in the choices a child might face. Such learning processes play obviously an important role in human development, and may also enable quick and effective application of robotic systems. Multi-level learning may indeed constitute a key line of research for HRI (Mavridis, 2015), which might again benefit from research in developmental psychology.

Reciprocally, the field of robotics provides interesting perspectives for psychologists, especially for research on atypical development. Atypical development might be a direct window on typical development and vice versa: “development is the key to understanding developmental disorders” (Karmiloff-Smith, 1998). Joint action and joint attention are for example usually impaired in children with ASD; the comparison with typical development has revealed different use of social gaze and often a lack of the declarative function, both for verbal and non-verbal communication. The exchanges between robotics and developmental psychology could help conceptualize the stages of joint attention in order to better understand how children develop joint attention and get through the whole sequence of declarative pointing. This will have an impact on elaborating intervention programs for children with neurodevelopmental disorders. Moreover, numerous intervention programs have recently been proposed showing the added value of therapy robot for the development of communication, play, or emotional skills (e.g., Robins et al., 2009; Huijnen et al., 2016).

In conclusion, the combination of insights and methods in robotics and developmental psychology allows researchers to conceive models of HRI in which the robots can come to develop motor, social, and cognitive skills. These models may benefit fundamental research on joint attention and joint action in typical development, but also early evaluation and intervention programs for atypical development (e.g., Dautenhahn, 2007). The continuation of these interdisciplinary discussions, which may possibly integrate some of the elements proposed in the present article, will undoubtedly lead to more and more solid HRI models in the next decades.

## Author Contributions

HC and MG devised the conceptual ideas presented in the article. HC drafted the manuscript. MG revised it critically and gave final approval of the version to be submitted.

## Conflict of Interest Statement

The authors declare that the research was conducted in the absence of any commercial or financial relationships that could be construed as a potential conflict of interest.

## Footnotes

1 For example, http://www.lucs.lu.se/epirob/
